# Analysis of risk factors of fungal superinfections in viral pneumonia patients: A systematic review and meta‐analysis

**DOI:** 10.1002/iid3.760

**Published:** 2022-12-31

**Authors:** Haiyang Ni, Hongying Yu, Qibin Lin, Jieying Zhong, Wenjin Sun, Hanxiang Nie

**Affiliations:** ^1^ Department of Respiratory & Critical Medicine Renmin Hospital of Wuhan University Wuhan Hubei China; ^2^ Department of infectious disease Ezhou Central Hospital Ezhou Hubei China

**Keywords:** corticosteroid, fungal infection, IPA, systematic review and meta‐analysis, viral pneumonia

## Abstract

**Background:**

Infections with fungi, such as *Aspergillus* species, have been found as common complications of viral pneumonia. This study aims to determine the risk factors of fungal superinfections in viral pneumonia patients using meta‐analysis.

**Objective:**

This study aims to determine the risk factors of fungal infection s in viral pneumonia patients using meta‐analysis.

**Methods:**

We reviewed primary literature about fungal infection in viral pneumonia patients published between January 1, 2010 and September 30, 2020, in the Chinese Biomedical Literature, Chinese National Knowledge Infrastructure, Wanfang (China), Cochrane Central Library, Embase, PubMed, and Web of Science databases. These studies were subjected to an array of statistical analyses, including risk of bias and sensitivity analyses.

**Results:**

In this study, we found a statistically significant difference in the incidence of fungal infections in viral pneumonia patients that received corticosteroid treatment as compared to those without corticosteroid treatment (*p* < .00001). Additionally, regarding the severity of fungal infections, we observed significant higher incidence of invasive pulmonary aspergillosis (IPA) in patients with high Acute Physiology and Chronic Health Evaluation (APACHE) II scores (*p* < .001), tumors (*p* = .005), or immunocompromised patients (*p* < .0001).

**Conclusions:**

Our research shows that corticosteroid treatment was an important risk factor for the development of fungal infection in patients with viral pneumonia. High APACHE II scores, tumors, and immunocompromised condition are also important risk factors of developing IPA. The diagnosis of fungal infection in viral pneumonia patients can be facilitated by early serum galactomannan (GM) testing, bronchoalveolar lavage fluid *Aspergillus* antigen testing, culture, and biopsy.

## Graphical abstract

Viruses are common pathogens of respiratory infections that can lead to severe morbidity and mortality. Viral pneumonia can be caused by virus invading human body. Infection with fungal pathogens has been shown as complications of viral pneumonia. Our research shows that corticosteroid treatment was an important risk of fungal infection, especially invasive pulmonary aspergillosis (IPA), in viral pneumonia patients. High Acute Physiology and Chronic Health Evaluation II scores, tumors, and immunocompromised condition, were also important risk factors of IPA inn viral pneumonia patients. We recommend that early testing for IPA, such as early serum galactomannan testing, bronchoalveolar lavage fluid Aspergillus antigen testing, culture, or biopsy should be prescribed to these patients to reduce potential mortality rate.

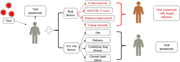

## 1 INTRODUCTION

1.1

Viruses are common pathogens of respiratory infections that can lead to severe morbidity and mortality.^1^ For example, Influenza virus has caused numerous periodic and/or seasonal epidemics or pandemics,^2^ resulting in significant death and disabilities. In the United States, up to 60,000 flu related deaths occurs every year.^3^ Worldwide, the mortality rate of the H5N1 pandemic in 2004–2005 is approximately 61%, and the hospitalized mortality rate of the H1N1 pandemic in 2009 reaches up to 46% in certain areas.^1^ More recently, coronavirus, particularly severe acute respiratory coronavirus‐2 (SARS‐CoV‐2), the causing agent of COVID 19, has caused severe global pandemic. For instance, as of November 24, 2022, over 600 million cases of SARS‐CoV‐2 infections has been confirmed worldwide since the appearance of the first case of SARS‐CoV‐2 pneumonia in December 2019. Additionally, over 1 million deaths caused by SARS‐CoV‐2 pneumonia, or its related complications, have been reported.^4^

Fungi exist widely in nature and are also part of the human microbiome. They usually do not cause diseases unless the host immune system is compromised or a dysbiosis of the host microbiota occurs.^5^ However, in recent years, fungal infections, especially opportunistic infections, have shown a significant upward trend.^5^ This is likely associated with the increased usage of immunosuppressive therapies, the lack of new antifungal drugs or vaccines, and the increased incidence of microbiota disorders due to antibiotic abuse. Although the incidence of invasive fungal infections is lower than that of superficial fungal infections, they often lead to a much high mortality rate.^6^ Therefore, there is a critical need to understand the risk factors and the etiology of the invasive fungal infections to prevent or develop novel therapeutics for these diseases. Despite that invasive infections can be caused by a variety of fungi, over 90% of fungal‐related deaths reported are caused by fungal species of the following four genera: *Cryptococcus*, *Candida*, *Aspergillus*, and *Pneumocystis*.^6^ Regarding the lung fungal infections, *Aspergillus* is one of the most common pathogen, especially in people with impaired immune response, such as those that received corticosteroid treatment, and often leads to the development of invasive pulmonary aspergillosis (IPA). IPA can result in a 100% fatality in patients with delayed diagnosis or treatment.^7^ Even with prompt diagnosis and intervention, the overall mortality rate of IPA remains as high as 50%.

Infection with fungal pathogens, such as the *Aspergillus* species, has been shown as complications of viral pneumonia, which can lead to increased morbidity and mortality.^8^, ^9^ However, the risk factors for fungal infections in viral pneumonia patients remain unclear. In the present study, we conducted a systematic review and meta‐analysis of published articles and identified key risk factors related to fungal infections post viral pneumonia. We also discussed recommendations for treating these complications.

## 2 MATERIALS AND METHODS

This study is a systematic review that is based on previously published data, thus the ethical approval and informed consent are not applicable.

### 2.1 Literature search strategy

The literature search of this study consisted of two steps. The first step was to search for all relevant Chinese‐language and English‐language literature published from January 1, 2010 to September 30, 2020 in electronic databases including Chinese Biomedical Literature (CBM), Chinese National Knowledge Infrastructure (CNKI), Cochrane Central Library, Embase, PubMed, Wanfang, and Web of Science. The keywords used to identify studies involving fungal infections in viral pneumonia patients were as follows: “Pneumonia, Viral,” “Pneumonias, Viral,” “Viral Pneumonia,” “Viral Pneumonias,” “Mycoses,” “Fungus Diseases,” “Disease, Fungus,” “Diseases, Fungus,” “Fungus Disease,” “Fungus Infections,” “Fungus Infection,” “Infection, Fungus,” “Infections, Fungus,” “Fungal Infections,” “Fungal Infection,” “Infection, Fungal,” “Infections, Fungal,” “Fungal Diseases,” “Disease, Fungal,” “Diseases, Fungal,” “Fungal Disease,” “Influenza,” “SARS‐COV1,” “SARS‐COV2,” “Aspergillosis,” “Pneumocystis Jirovecii,” and “Mucormycosis.” The literature searches were not restricted by language, country, and journal of publication. Next, we refined the literatures coming out of the searches by examining their titles and abstracts to exclude studies that are not related to either viral pneumonia or fungal infection. Afterwards, we further filtered the remaining literatures by reviewing the full content of the studies to confirm fungal infection was always acquired after viral pneumonia. The second step was a manual search of the reference lists in the retrieved original articles.

### 2.2 Study selection criteria

To be considered for inclusion in the meta‐analysis, the studies have to fulfill the following criteria: (1) the patients reported in the study were diagnosed with viral pneumonia and fungal infection acquired after viral pneumonia; (2) the studies need to report one or more of the following factors: age, histories of other diseases including diabetes, chronic heart failure, chronic kidney disease, non‐pneumonia lung diseases, and cancer, immune status, usage of corticosteroid treatment, and Acute Physiology and Chronic Health Evaluation (APACHE) II admission score. The exclusion criteria were as follows: (1) duplicated publications showing in multiple databases; (2) review articles; (3) studies that include less than five cases; (4) the patients reported do not have viral pneumonia or fungal infection; (5) papers with incomplete outcome data or for which the full text could not be obtained; (6) the patients reported in the study included children and infants.

### 2.3 Data collection

According to the inclusion and exclusion criteria two investigators independently screened the titles, abstracts, and full text of studies using a standard form to identify eligible studies. After that, the data from each included study were extracted by the above two researchers. Any conflicts were checked by a third researcher, settled with discussion and consensus sought. The following data from each article were extracted: first author, year of publication, year, country, and size of study, age, population, diagnostic methods for viral pneumonia, species of virus, diagnostic methods for fungal infections, species of the fungi, laboratory test results, and disease outcomes.

### 2.4 Disease definitions and outcomes

Viral pneumonia was defined as at least one positive result in virus testing, such as real‐time reverse transcription‐polymerase chain reaction, viral isolation,^10^ serological testing, and rapid influenza diagnostic test (RIDT), along with new or progressive radiographic infiltration. If radiological signs were not mentioned in the article, patients who were hospitalized, or treated in Intensive Care Unit (ICU), Respiratory Intensive Care Unit (RICU) or Mobile Intensive Care Unit (MICU) were also defined to have viral pneumonia. Fungal infection was defined as culture, biopsy, or galactomannan (GM) detection positive (>0.5), regardless of the infection site. A low‐to‐moderate corticosteroid dose was defined as 25–150 mg/d methylprednisolone or equivalent drug/dose, whereas a high corticosteroid dose was defined as greater than 150 mg/d ethylprednisolone or equivalent drug/dose.^11^ Outcomes were risk factors of viral pneumonia patients with fungal infection. The primary outcome measure was corticosteroid treatment, and secondary outcome measures were age, APACHE II score, diabetes, chronic heart failure, chronic kidney disease, underlying lung disease, cancer disorder and immunosuppression.

### 2.5 Risk of bias assessment

Given that all studies included in the meta‐analysis are cross‐sectional studies, thus two researchers used the Agency for Healthcare Research and Quality (AHRQ) scale to assess the methodology and quality of these studies. Any conflicts were checked by a third researcher, settled with discussion and consensus sought. The AHRQ scale contains 11 parameters that are used for scoring. The score of each parameter ranges between 0 and 1 with 1 represents the highest quality and lowest risk of bias.

### 2.6 Statistical analysis

The sensitivity analysis was performed using Stata v12.0 software, whereas other statistical analyses were performed using RevMan 5.3 software. The *I* ^2^ test was used to measure the heterogeneity of the studies. A low heterogeneity was defined as *I* ^2^ < 50%. For studies with low heterogeneity, a fixed‐effects model was used for statistical analysis. A high heterogeneity was defined as *I* ^2^ > 50%. For studies with I^2^ > 50%, a sensitivity analysis was performed to assess the stability of the result of the meta‐analysis and to determine the source of heterogeneity. If a significant heterogeneity was not identified, a random‐effects model was adopted for the statistical analysis. Standardized mean difference was used to establish effect quantity for continuous data, and risk ratio (RR) was used for describing dichotomous data. Potential publication bias was assessed by funnel plots. Test for publication bias was not performed in less than five of the included articles. In addition, for studies including corticosteroid treatment, we also conducted a subgroup analysis based on the dose used.

## 3 RESULTS

### 3.1 Literature search results

**Figure 1:**
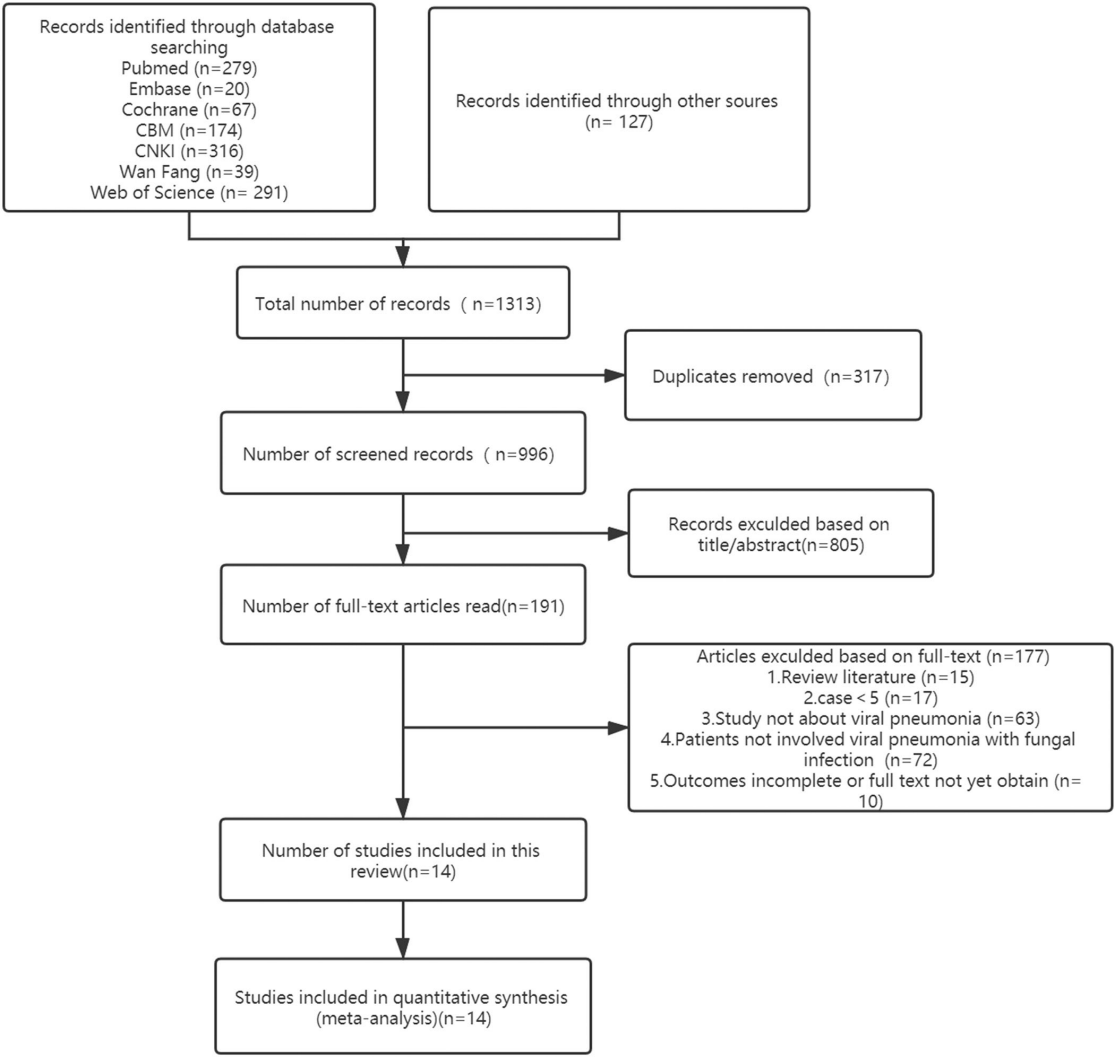
Flow chart

The initial literature search resulted in a total of 1313 studies from all the databases used and their distributions were as follows: CBM (*n* = 174), CNKI (*n* = 316), Cochrane Central Library (*n* = 67), Embase (*n* = 20), PubMed (*n* = 279), Wanfang (*n* = 39), Web of Science (*n* = 291), and other sources (*n* = 127). We then removed 317 duplicated articles and the remaining ones were filtered based on the inclusion and exclusion criteria. This reduced the total article number to 191 articles, which were then subjected to screening of titles, abstracts, or both. Finally, after further reviewing the full content of the articles, a total of 14 articles were included in this meta‐analysis (Figure 1).

### 3.2 Study characteristics

The characteristics of included studies were summarized in Table 1. All studies followed cross‐sectional design and were conducted from 2007 to 2019. Of these, 8 were conducted solely in China, 1 in Korea, 1 in United States, 1 in Canada, 1 in Belgium, and 2 were multicenter studies that were conducted across multiple countries in Europe. The sample sizes of the studies ranged from 8 to 2141. All reported the species of virus and fungus responsible for the infections. Thirteen studies described the diagnostic methods for the virus, and 12 studies described the diagnostic methods for the fungus (Table 1). Features associated with corticosteroid treatment and other outcomes among viral pneumonia patients in these studies are summarized in Tables 2 and 3.

**Table 1:** Baseline characteristics of the included studies

Author, year, reference		Country	Age (year)	Population	Diagnostic methods of virus	species of virus	Diagnostic methods of fungi	species of Fungus	Outcomes studied	AHRQ
Wang et al. 2010^12^		China	38 ± 20	Critical patients	RT‐PCR	H1N1	Culture	*Candida albicans*/*Aspergillus* fumigatus	Corticosteroid treatment	9
Yang et al. 2018^13^		China	56 (Median)	RICU	RT‐PCR	H1N1	BALF culture	*Aspergillus*/C. *Candida parapsilosis*	Corticosteroid treatment	7
Cao et al. 2016^10^		China	58 (Median)	Hospitalization	RT‐PCR/viral isolation/erological testing	H7N9	SARS A	*Aspergillus*/mucor/*Candida albicans*	Corticosteroid treatment	10
Kim et al. 2011^14^	Corticosteroids	Korea	57 ± 17	ICU	RT‐PCR	H1N1	NA	*Aspergillus*/*Candida albicans*	Corticosteroid treatment	8
	No Corticosteroids		54 ± 19							
Li et al. 2017^15^		China	34.4 (median)	Hospitalization	RT‐PCR	H1N1	Culture/A	Invasive fungal	Corticosteroid treatment	9
Martin‐Loeches et al. 2011^16^		Europe (ESICM)	43	ICU	RT‐PCR/culture	H1N1	NA	*Aspergillus*	Corticosteroid treatment	8
Crum‐Cianflone 2016^9^		USA	66	ICU	RT‐PCR/culture	H1N1, H3N2, Influenza B virus	Culture/A	*Aspergillus*	Corticosteroid treatment	8
Wauters et al. 2012^17^		Belgium	49 ± 14	ICU	RT‐PCR	H1N1	BALF culture/biopsy/GM/A	*Aspergillus*	Multiple risk factors	9
Huang et al. 2015^18^		Taiwan, China	66 ± 19	Hospitalization	RT‐PCR	Influenza A virus	Culture	Yeast	Corticosteroid treatment	8
Ku et al. 2017^19^		Taiwan, China	65	ICU	RT‐PCR/viral isolation/RIDT	H1N1, H3N2, other Influenza A virus	BALF culture/biopsy/GM/A	*Aspergillus*	Multiple risk factors	9
Schwartz et al. 2020^11^		Canada	56 ± 16	ICU	RT‐PCR/culture	H1N1, H3N2, Influenza B virus	BALF culture/biopsy/GM/A	*Aspergillus*	Multiple risk factors	7
Huang et al. 2019^20^	IPA	China	63 ± 13	MICU	RT‐PCR/viral isolation/erological testing	Influenza	BALF culture/biopsy/GM/A	*Aspergillus*	Multiple risk factors	8
	No IPA		57 ± 18							
Schauwvlieghe et al. 2018^21^		Europe (Multi‐center, two in Belgium, five in the Netherlands)	59 ± 15	ICU	RT‐PCR	Influenza	BALF culture/biopsy/GM/A	*Aspergillus*	Multiple risk factors	9
Zou et al. 2020^22^		China	57 (median)	Hospitalization/ICU	NA	H7N9	A	*Aspergillus*	Multiple risk factors	8

*Note*: Data are expressed as the mean ± standard deviation unless otherwise stated.

Abbreviations: A, Invasive pulmonary aspergillosis and mucormycosis were diagnosed in accordance with the revised definitions of invasive fungal diseases from the European Organization for Research and Treatment of Cancer/Invasive Fungal Infections Cooperative Group and the National Institute of Allergy and Infectious Diseases Mycoses Study Group Consensus Group. RT‐PCR: the real‐time reverse transcription‐polymerase chain reaction; AHRQ, Agency for Healthcare Research and Quality; BALF, bronchoalveolar lavage fluid; ESICM, Data were obtained from a voluntary registry instituted by the European Society of Intensive Care Medicine; H1N1, hemagglutinin 1 neuraminidase 1; ICU, intensive care units; MICU, medical intensive care unit; NA, not available; RICU, respiratory intensive care unit; RIDT, rapid influenza diagnostic tests; SARS, severe acute respiratory syndrome.

**Table 2:** Effects of corticosteroid treatment in the included studies

Author, year, reference	Corticosteroid treatment				Corticosteroid treatment		Corticosteroid treatment			
	Yes		No		Corticosteroid dose	Corticosteroid dose grade	Yes		No	
	Total population	Fungal infection	Total population	Fungal infection			Total population	*Aspergillus* infection	Total population	*Aspergillus* infection
Wang et al. 2010^12^	30	6	25	6	1–3 mg/kg/d	A	NA		NA	
Yang et al. 2018^13^	5	2	3	0	40–80 mg/d	A	5	1	3	0
Cao et al. 2016^10^	65	6	65	4	≤80 mg/d (IQR, 40–120 mg)	A	65	4	65	4
Kim et al. 2011^14^	65	5	65	1	75 mg/d (IQR, 50–81 mg)	A	65	2	65	0
Li et al. 2017^15^	1055	25	1086	3	NA	A 662^a^ B 367	NA		NA	
Martin‐Loeches et al. 2011^16^	126	3	94	1	NA	NA	126	3	94	1
Crum‐Cianflone 2016^9^	2	1	6	4	75 mg/d	A	2	1	6	4
Wauters et al. 2012^17^	14	7	26	2	800 mg/7days	A	14	7	26	2
Huang et al. 2015^18^	22	2	10	1	NA	NA	NA		NA	
Ku et al. 2017^19^	40	16	10	5	NA (cumulative doses)	NA	40	16	10	5
Schwartz et al. 2020^11^	67	4	40	4	≥150 mg/d	B	67	4	40	4
Huang et al. 2019^20^	38	29	71	34	NA (cumulative doses)	NA	38	29	71	34
Schauwvlieghe et al. 2018^21^	145	46	281	36	0.14 mg/kg/d (0.06–0.28)	A	145	46	281	36
Zou et al. 2020^22^	251	15	84	3	80 mg/d	A	251	15	84	3

*Note*: Corticosteroid dose: A low‐to‐moderate corticosteroid dose was defined as 25–150 mg/d methylprednisolone or its equivalent, whereas a high corticosteroid dose was defined as greater than 150 mg/d ethylprednisolone or its equivalent.

Abbreviations: A, low‐to‐moderate corticosteroid dose; B, high corticosteroid dose.

^a^ Nine people were infected with fungus at low to‐moderate corticosteroid dose, and 16 were infected with fungus at high corticosteroid dose.

**Table 3:** Other outcomes in the included studies

Author, year, reference		Age (years)	APACHE II admission	Diabetes, *n*	Chronic heart failure, *n*	Chronic kidney disease, *n*	Underlying lung disease, *n*	Cancer disorder, *n*	Immunocompromised, *n*
						Control group	Intervention group	Control group	Intervention group
Wauters et al. 2012^17^	IPA (*n* = 9)	53 ± 10	25 ± 8	1	1	0	1	NA	NA
	No IPA (*n* = 31)	48 ± 14	23 ± 9	4	3	1	1	NA	NA
Ku et al. 2017^19^	IPA (*n* = 21)	NA	NA	7	NA	NA	2	5	NA
	No IPA (*n* = 29)	NA	NA	12	NA	NA	7	1	NA
Schwartz et al. 2020^11^	IPA (*n* = 8)	51 ± 10	25 ± 6	NA	NA	NA	4	NA	1
	No IPA (*n* = 103)	57 ± 16	24 ± 9	NA	NA	NA	39	NA	18
Huang et al. 2019^20^	IPA (*n* = 63)	63 ± 13	NA	19	9	2	13	15	15
	No IPA (*n* = 46)	57 ± 18	NA	12	3	2	5	3	3
Schauwvlieghe et al. 2018^21^	IPA (*n* = 83)	60 ± 12	25 ± 9	10	NA	16	13	26	22
	No IPA (*n* = 349)	59 ± 16	22 ± 7	78	NA	55	66	61	32
Zou et al. 2020^22^	IPA (*n* = 18)	61 ± 13	NA	4	2	0	2	NA	0
	No IPA (*n* = 317)	56 ± 16	NA	53	30	12	14	NA	5

*Note*: Data are expressed as the mean ± standard deviation unless otherwise stated.

Abbreviations: APACHE II, Acute Physiology and Chronic Health Evaluation II; IPA, invasive pulmonary aspergillosis.

### 3.3 Risk of bias in the included studies

The risk of bias of the studies analyzed in the present meta‐analysis were summarized in Table 1. All studies were rated as having low‐to‐moderate risk of bias.

### 3.4 Outcome

#### 3.4.1 Primary outcome

##### Corticosteroid treatment

**Figure 2:**
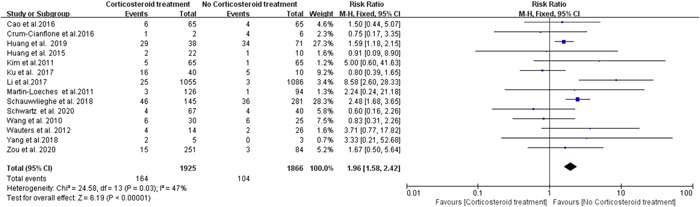
Forest plot of risk of fungus infection during corticosteroid treatment for viral pneumonia

**Figure 3:**
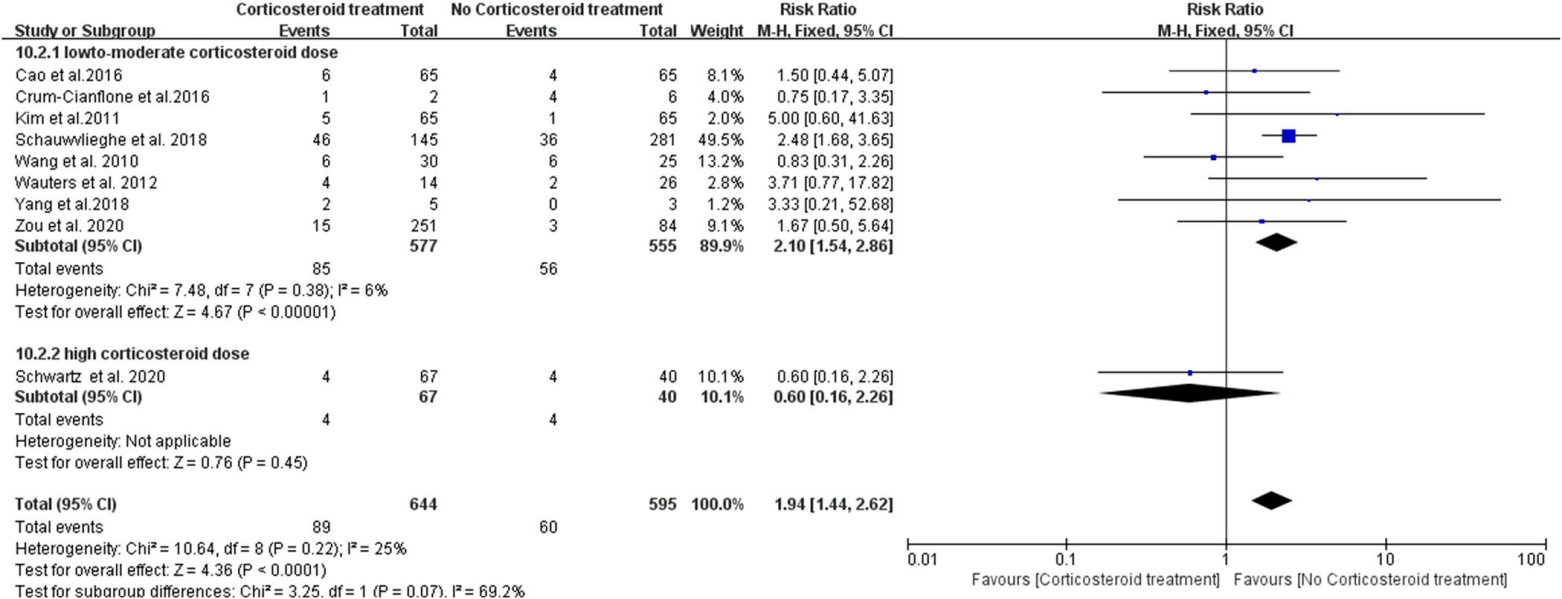
Subgroup analysis forest plot of risk of fungus infection during corticosteroid treatment for viral pneumonia

**Figure 4:**
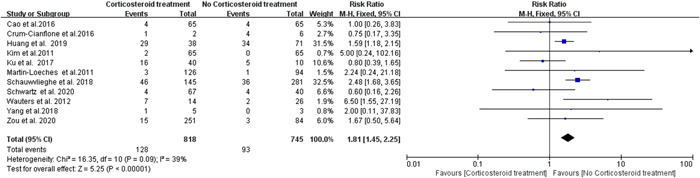
Forest plot of risk of aspergillus infection during corticosteroid treatment for viral pneumonia

Fourteen studies reported increased risk of fungal infection when using corticosteroid treatment for viral pneumonia. We did not observe significant heterogeneity (*I* ^2^ = 47%) among these studies and therefore the fixed‐effects model was employed. A significant difference in the incidence of fungal infection in patients with viral pneumonia was observed between the corticosteroid treated group and the untreated group (RR = 1.96; 95% confidence interval [CI] 1.58–2.42; *p* < .00001; Figure 2). According to the funnel plot, no publication bias was observed in the present meta‐analysis (Supporting Information: Figure 1). Since the *I* ^2^ value (47%) is close to 50%, we further conducted subgroup analysis based on the dose of corticosteroids used in these studies. Specifically, we divided the corticosteroid treated group into two subgroups: the low‐to‐moderate dose group and the high dose group (see Section Objective, 2). Interestingly, we observed a significant difference between the low‐to‐moderate corticosteroid treated group and untreated group (RR = 2.10; 95% CI 1.54–2.86; *p* < .00001; *I* ^2^ = 6%; Figure 3), whereas no significant statistical difference was found between the high dose treated group versus the untreated group (RR = 0.60; 95% CI 0.16–2.26; *p* = .45; Figure 3). Eleven studies reported the risk of *Aspergillus* infection during corticosteroid treatment for viral pneumonia. No significant heterogeneity (*I* ^2^ = 39%) was observed and thus the fixed‐effects model was employed. This revealed a significant difference between the corticosteroid treatment group and no corticosteroid treatment (RR = 1.81; 95% CI 1.45–2.25; *p* < .00001; *I* ^2^ = 39%; Figure 4) in the incidence of Aspergillosis. Additionally, no publication bias was observed in the present meta‐analysis (Supporting Information: Figure 2).

### 3.5 Secondary outcomes

Three studies reported the risk of IPA infection during the viral pneumonia in cancer disorder. While, sensitivity analysis shown that Schwartz et al. 202011 had a significant heterogeneity (Supporting Information: Figure 4). Therefore, we excluded this study in the statistics analyze of the risk of IPA infection during the viral pneumonia in cancer disorder. The results showed a significant difference between the IPA group and no IPA group in the risk of APACHE II score (RR = 0.36; 95% CI 0.14–0.58; *p* < .001; *I* ^2^ = 0%; Supporting Information: Figure 3A), cancer disorder (RR = 4.29; 95% CI 1.55–11.83; *p* = .005; *I* ^2^ = 0%; Supporting Information: Figure 3B), and immunosuppression (RR = 2.69; 95% CI 1.73–4.17; *p* < .0001; *I* ^2^ = 0%; Supporting Information: Figure 3C). While, there was no significant difference between the IPA group and no IPA group in the risk of age, diabetes, chronic heart failure, chronic kidney disease, underlying lung disease (*p* > 0.05; Supporting Information: 3D–H).

### 3.6 Publication bias

No significant visual asymmetry was observed in funnel plots. According to the funnel plot, there was no publication bias in these studies (Supporting Information: Figures [Link], [Link], [Link]).

### 3.7 Sensitivity analysis

As mentioned above, we identified that the study in one article, Schwartz et al.,^11^ showed a significant heterogeneity as determined by the sensitivity analysis. For all other studies, the pooled effect did not change significantly after excluding each study individually (Supporting Information: Figures [Link], [Link], [Link], [Link], [Link], [Link], [Link]).

## 4 DISCUSSION

The purpose of this systematic review and meta‐analysis was to identify the risk factors for fungal infection in patients with viral pneumonia. We found that corticosteroid treatment is an important risk factor (*p* < .00001). Severe respiratory virus infections, such as viral pneumonia, can cause excessive inflammatory responses, which may lead to the production of cytokine storm, resulting in acute respiratory distress syndrome (ARDS) and other life‐threatening disease conditions.^23^, ^24^ Additionally, inflammatory cytokines can inhibit the hypothalamic pituitary‐adrenal axis, causing adrenal insufficiency and further worsening the disease.^25^ Corticosteroids can reduce systemic inflammation by inhibiting inflammatory cell proliferation and cytokine production.^26^, ^27^ In fact, corticosteroids have been shown to improve the immune‐homeostasis of patients with septic shock through this mechanism.^28^ Consequently, corticosteroids have been used to treat severe viral pneumonia and its complication ARDS.^16^ However, whether corticosteroids should always be used for viral pneumonia treatment was still under debate. A meta‐analysis by Lansbury et al. suggested that corticosteroid treatment increases the mortality of patients with viral pneumonia.^29^ Another study showed that early use of glucocorticoids was a risk factor for critical disease and death from pH1N1 infection.^29^ We recommend that guidelines on glucocorticoid use be established and enforced. On the contrary, another meta‐analysis of severe pneumonia showed that systemic corticosteroid treatment can reduce the mortality rate in adult patients.^30^ One other report has also suggested that long‐term glucocorticoid therapy is safe and beneficial for patients with ARDS.^26^ Similar observations have been reported by Quispe‐Laime et al., which showed that prolonged low‐dose corticosteroid treatment for viral pneumonia patients reduces the mortality rate.^31^ Additionally, a recent study of COVID‐19 also showed that oral or intravenous administration of dexamethasone (at a dose of 6 mg, once a day) significantly improved the disease outcome and reduced mortality rate in COVID19 patients with mechanical ventilation.^32^ Despite the controversy, many patients with severe pneumonia are still receiving corticosteroid treatment. For instance, during the 2009 H1N1 pandemic, up to 69% of critically ill patients were received corticosteroid treatment.^29^ Our analysis indicated that corticosteroid treatment is an important risk of fungal infection, especially IPA (*p* < .00001), in patients with viral pneumonia. It is known that the defense mechanisms for fungal infection of the human body include both innate and adaptive immunity. It has been demonstrated that the first responders of the innate immune response to fungal infection are phagocytes, and macrophages represent the major phagocytic cells.^33^, ^34^, ^35^ Pattern recognition receptors on macrophages can identify some surface structures of the micro‐organisms in a nonadaptive way to activate macrophages for participation in the immune regulation process.^36^ Fungal Mannan is a key surface structure of fungi that can be recognized by mannan‐binding proteins on phagocytes, allowing recognition and efficient phagocytosis even in the absence of opsonized complements or Igs.^35^ After recognizing and engulfing pathogens, macrophages can present antigens to T cells and secrete cytokines like IL‐10, TGF‐β, and IL‐4 to initiate immune responses including contributing to macrophage infiltration, enhancing phagocytic functions, triggering adaptive responses, and so on.^37^ Moreover, adaptive immunity was also involved in the immune response to fungi. It has been shown that an adaptive immune response dependent on T‐helper 1 (Th1) cells could mediate protection against fungal infections, while Th2 cell responses may terminate the protective response to infection.^38^ However, corticosteroid treatment can reduce the numbers of monocytes, macrophages and lymphocytes and change the distribution of inflammatory cells in the body.^39^ Forthermore, corticosteroid treatment has a strong ability to inhibit the activation, proliferation and migration of T cells and reduce the production of cytokines. In addition, corticosteroid treatment can also inhibit the cytotoxic function of natural killer cells, induce the production of Th2 cytokines, disrupt the balance of Th1/Th2 cells, and inhibit the function of phagocytes, which can accelerate the process of fungal infections.^39^ As such, these findings suggest that corticosteroid treatment can cause immunosuppression in patients to reduce the anti‐fungal infection ability of the human body. In addition, corticosteroid treatment can induce or aggravate the occurrence of fungal infections by changing the biological characteristics of fungi. Treatment with large doses of corticosteroids could promote the proliferation of *Aspergillus fumigatus* and *Aspergillus flavus* in vitro, stimulate the synthesis and release of various toxins, and enhance the invasiveness of *A. fumigatus*.^40^ Therefore, once patients develop immune dysfunction and lung injury during corticosteroid treatment for viral pneumonia, fungal infection is likely to occur.^40^ Surprisingly, our subgroup analysis suggested that higher fungal infection rate was only observed in low‐to‐moderate dose corticosteroid treatment group but not in high‐dose group when comparing to the untreated control. Future studies are warranted to understand the mechanism underlying this dose dependent differences.

IPA often occurs in patients with immune dysfunction and is known as an independent risk factor of death in critically ill patients.^41^ Early diagnosis and treatment of IPA has been shown to improve the prognosis.^42^ However, the clinical manifestations of IPA lack specificity and can show similar symptoms compared to other infections. Additionally, when IPA occurs along with viral pneumonia infection, the lung imaging progress of IPA may be incorrectly attributed to viral pneumonia or ARDS, thereby delaying the diagnosis and/or treatment of IPA.^43^ Our present research suggested that fungal infections, especially IPA, need to be considered when viral pneumonia patients were treated with corticosteroid. When pulmonary infiltrations were observed in these patients, IPA infection should be considered, and appropriate examinations should be performed. For example, early serum GM test, BALF for *Aspergillus* antigen detection, culture, or biopsy can be prescribed to the patient to facilitate diagnosis. Importantly, it is suggested that these patients to be tested for IPA, and if positive, begin appropriate antifungal therapy within 24–48 h of ICU admission.^43^

Further, our research also showed that viral pneumonia patients with high APACHE II scores, tumors, and immunocompromised condition should also be closely monitered for potential IPA infection, and the above‐mentioned examinations should be performed in a timely fashion. Finally, as cases of COVID‐19 are rising rapidly, this meta analysis will need to be updated in the future with the new SARS CoV‐2 and fungal superinfection data.

## 5 CONCLUSION

Our research shows that corticosteroid treatment was an important risk of fungal infection, especially IPA, in viral pneumonia patients. High APACHE II scores, tumors, and immunocompromised condition, were also important risk factors of IPA inn viral pneumonia patients. We recommend that early testing for IPA, such as early serum GM testing, BALF *Aspergillus* antigen testing, culture, or biopsy should be prescribed to these patients to reduce potential mortality rate.

Haiyang Ni: 469589313@qq.com

Hongying Yu: 1084951159@qq.com

Qibin lin: 576047617@qq.com

Jieying Zhong: zhongjieying123@163.com

Wenjin Sun: 927818674@qq.com

## AUTHOR CONTRIBUTIONS

Nie Hanxiang, Ni Haiyang, and Yu Hongying conceptualized and designed this study. Ni Haiyang and Yu Hongying performed the research and wrote the article. Ni Haiyang, Zhong Jieying, Lin Qibin, and Sun Wenjin collected the data. Nie Hanxiang approved the submission of the manuscript.

## ETHICS STATEMENT

All the authors agreed to submit the manuscript to Current Medical Science.

## Supporting information

Suppoporting information.

Suppoporting information.

Suppoporting information.

Suppoporting information.

Suppoporting information.

Suppoporting information.

Suppoporting information.

Suppoporting information.

Suppoporting information.

Suppoporting information.

Suppoporting information.

Suppoporting information.

Suppoporting information.

Suppoporting information.

## Data Availability

The data that support the findings of this study are available from the corresponding author upon reasonable request.
