# Supplementary figures and images for: Analysis of risk factors of fungal superinfections in viral pneumonia patients: A systematic review and meta‐analysis
Source: Immun Inflamm Dis. 2022 Dec 31;11(1):e760. doi: 10.1002/iid3.760 (PMC9804449; doi:10.1002/iid3.760)

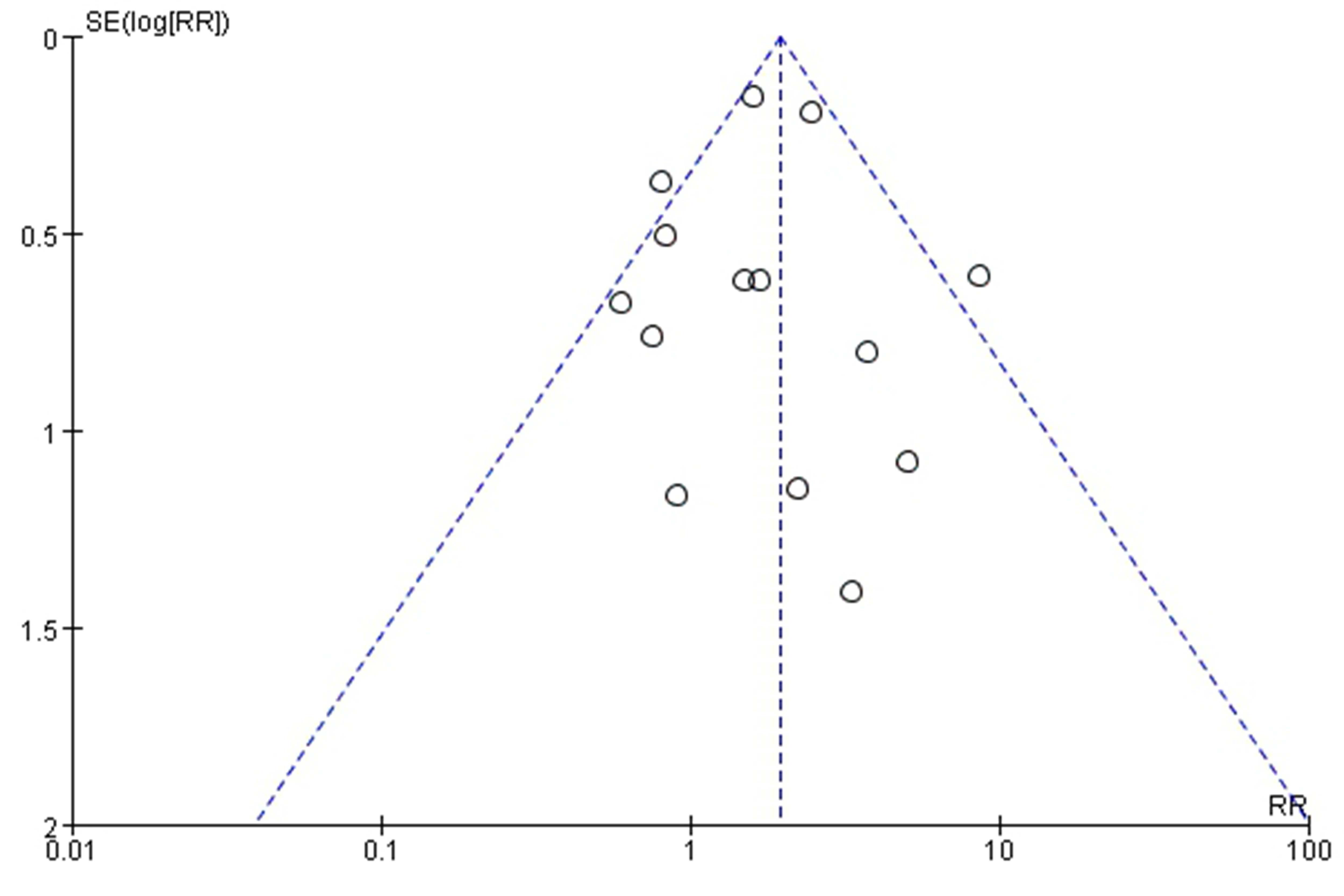

Supplement: Supplementary file 1 — Suppoporting information. [file IID3-11-e760-s013.jpg]

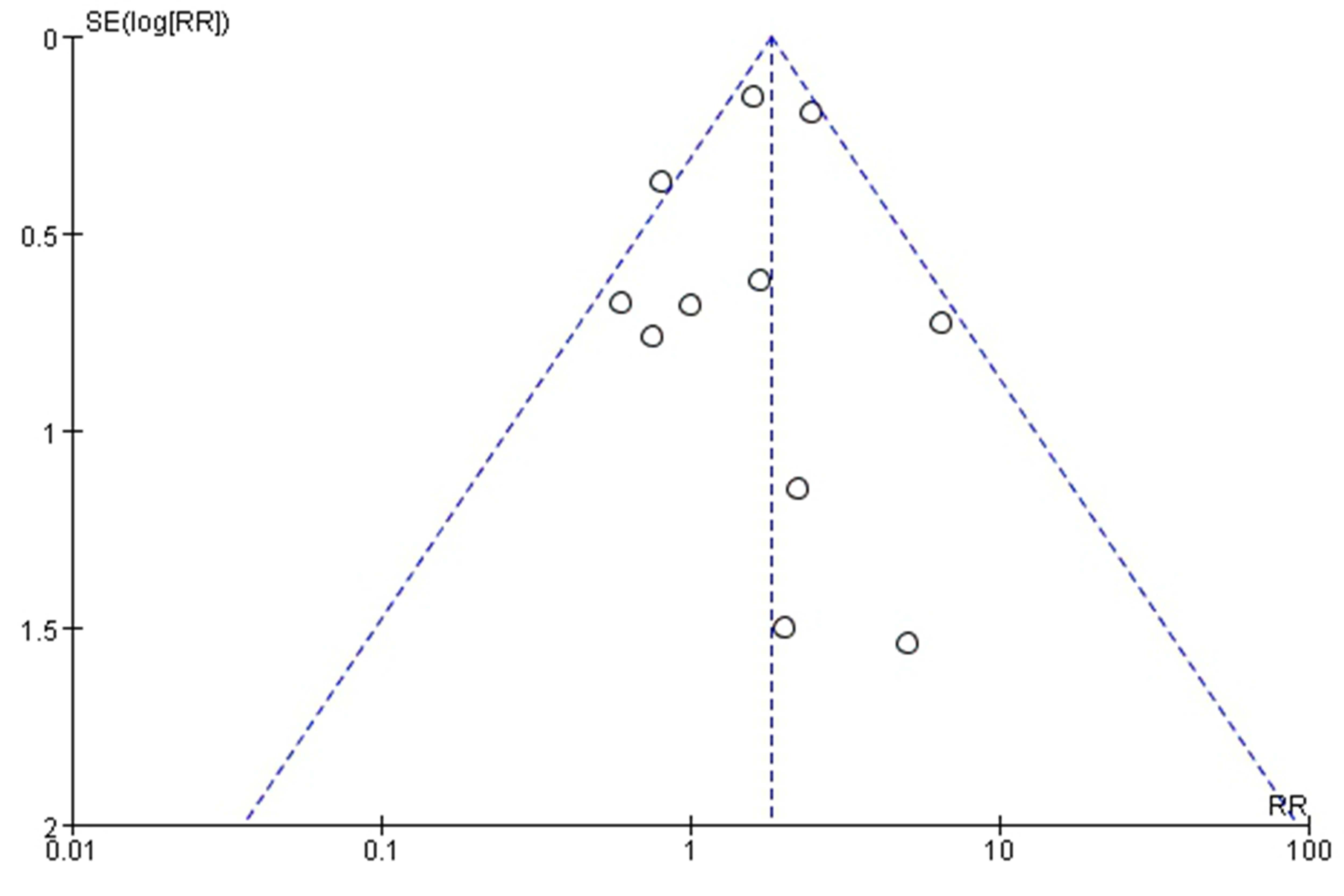

Supplement: Supplementary file 2 — Suppoporting information. [file IID3-11-e760-s012.jpg]

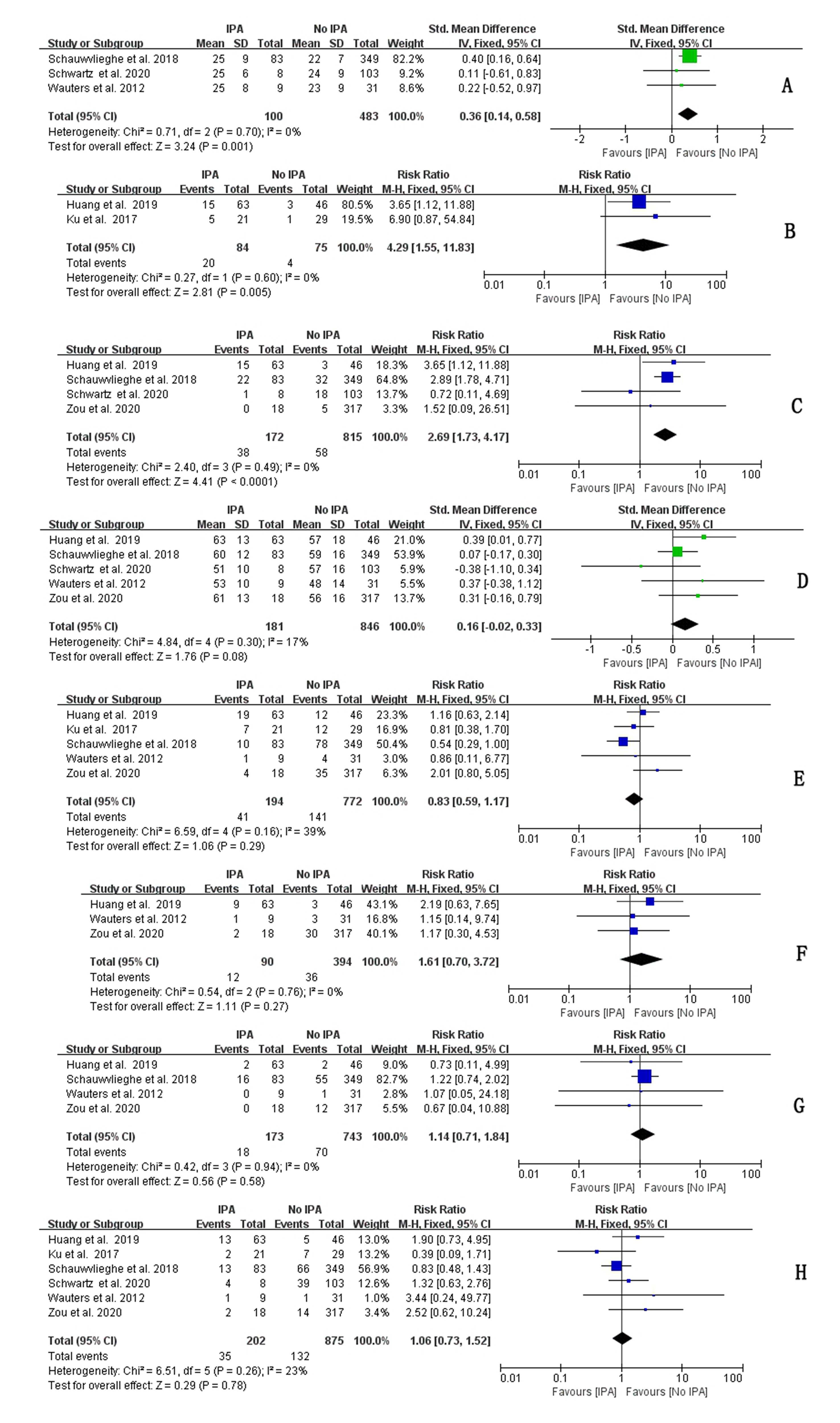

Supplement: Supplementary file 3 — Suppoporting information. [file IID3-11-e760-s011.jpg]

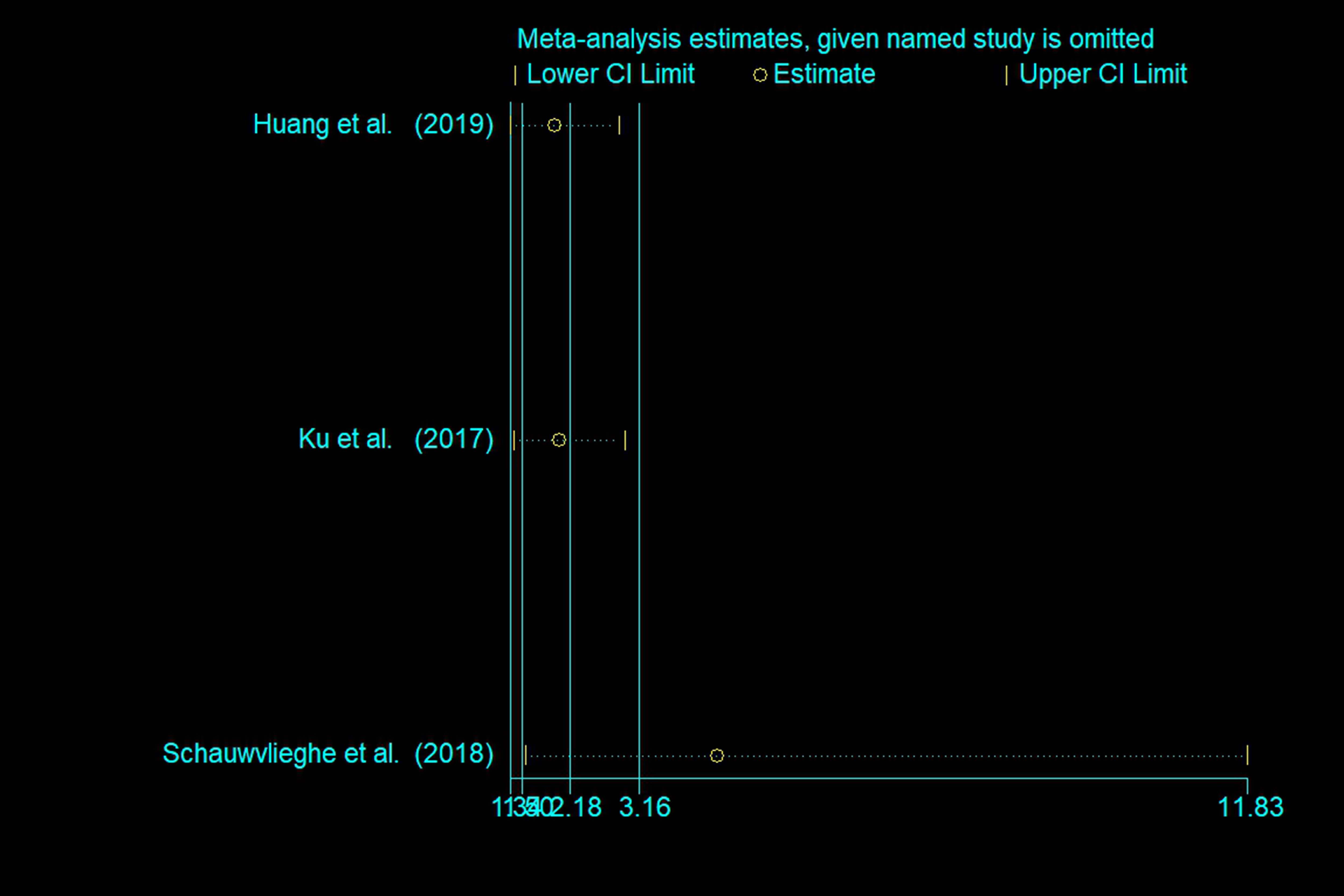

Supplement: Supplementary file 4 — Suppoporting information. [file IID3-11-e760-s014.jpg]

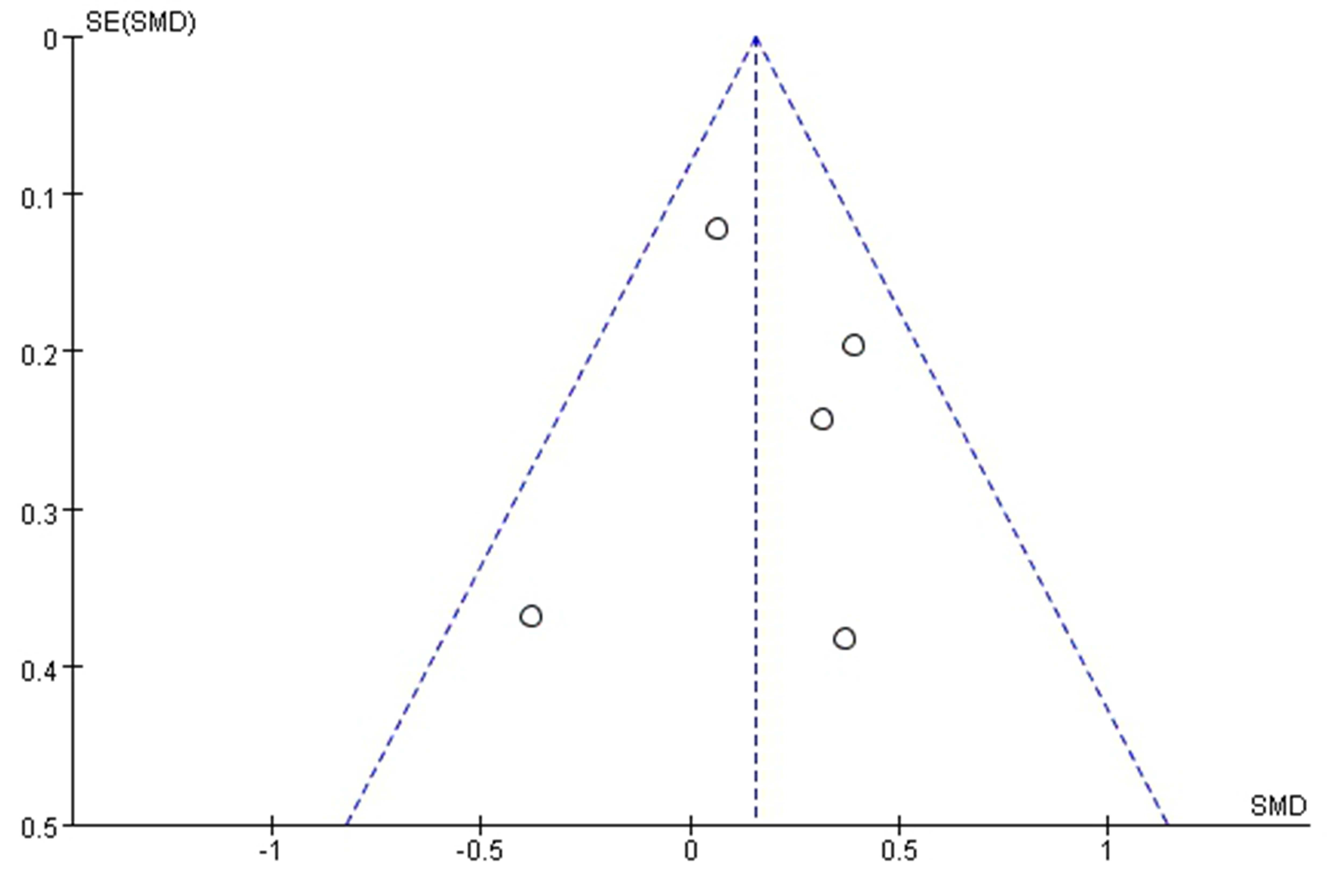

Supplement: Supplementary file 5 — Suppoporting information. [file IID3-11-e760-s002.jpg]

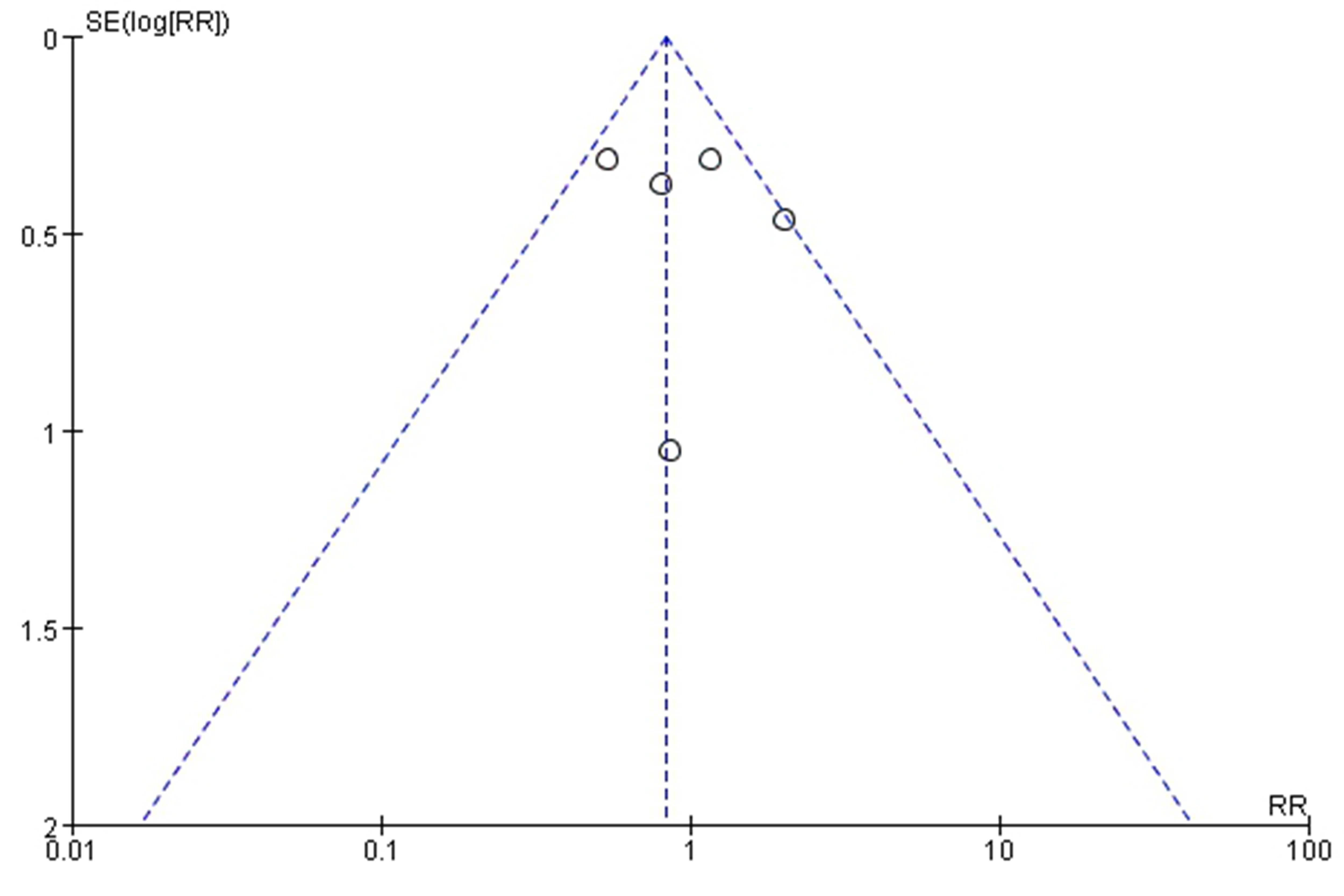

Supplement: Supplementary file 6 — Suppoporting information. [file IID3-11-e760-s007.jpg]

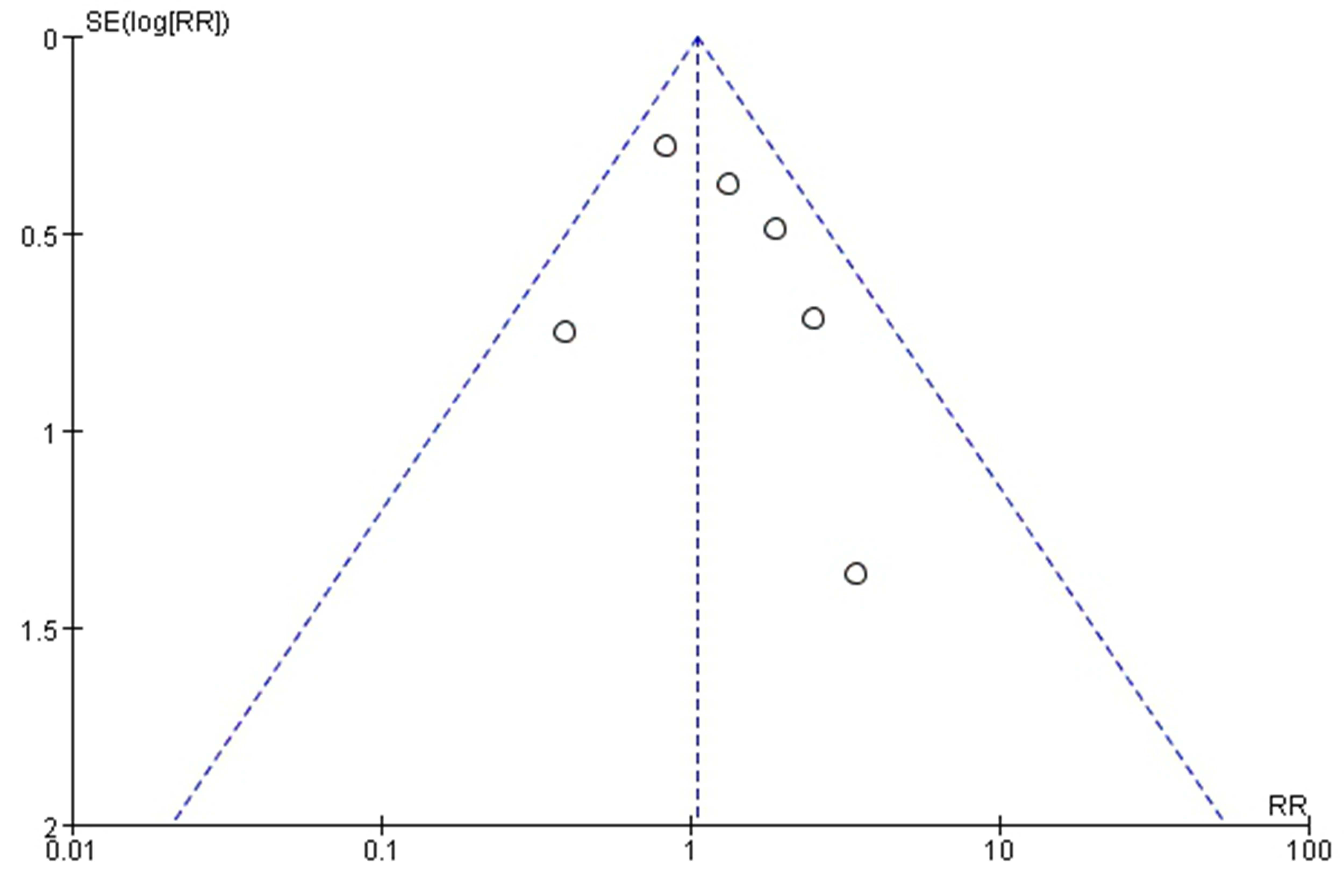

Supplement: Supplementary file 7 — Suppoporting information. [file IID3-11-e760-s009.jpg]

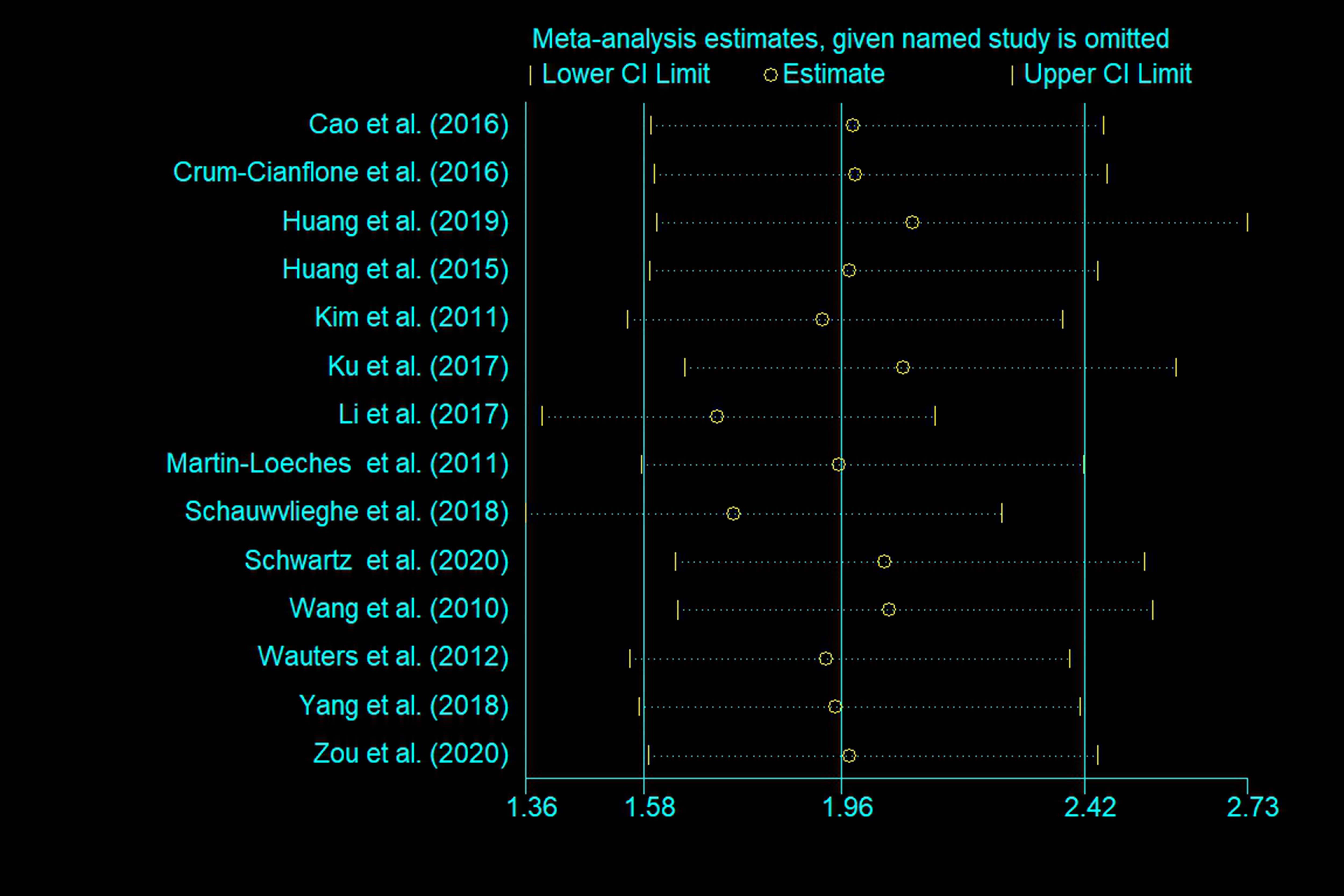

Supplement: Supplementary file 8 — Suppoporting information. [file IID3-11-e760-s010.jpg]

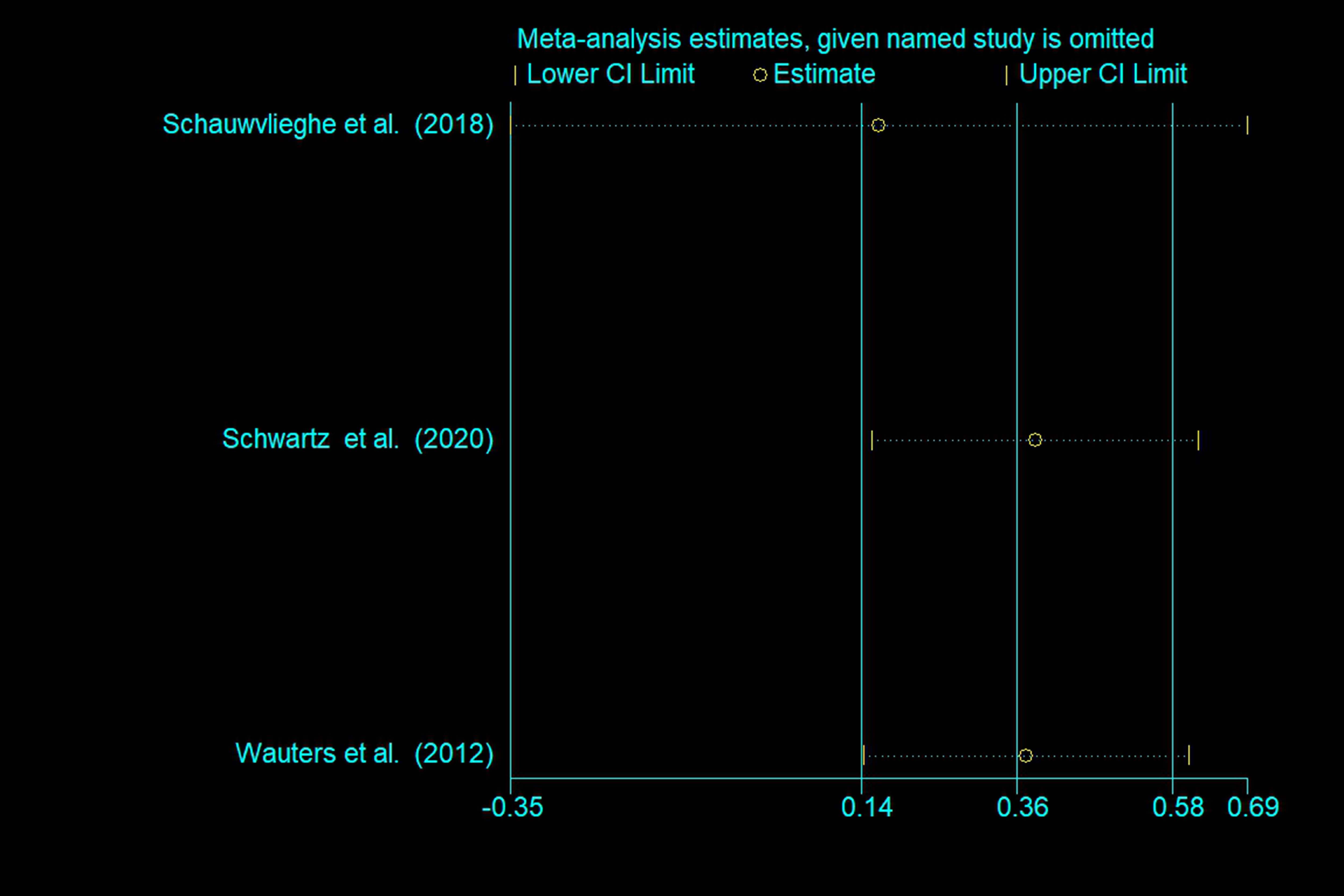

Supplement: Supplementary file 9 — Suppoporting information. [file IID3-11-e760-s008.jpg]

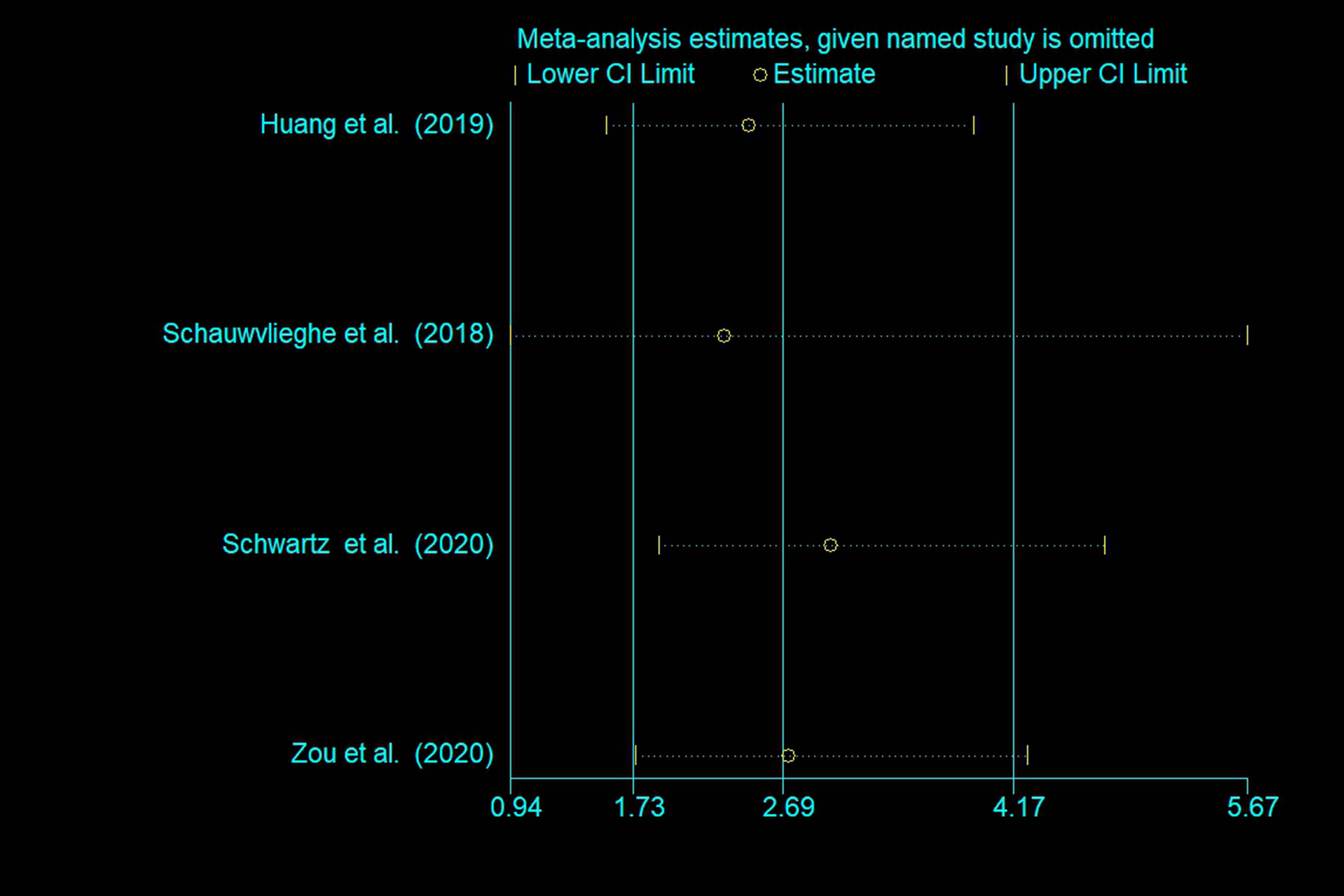

Supplement: Supplementary file 10 — Suppoporting information. [file IID3-11-e760-s005.jpg]

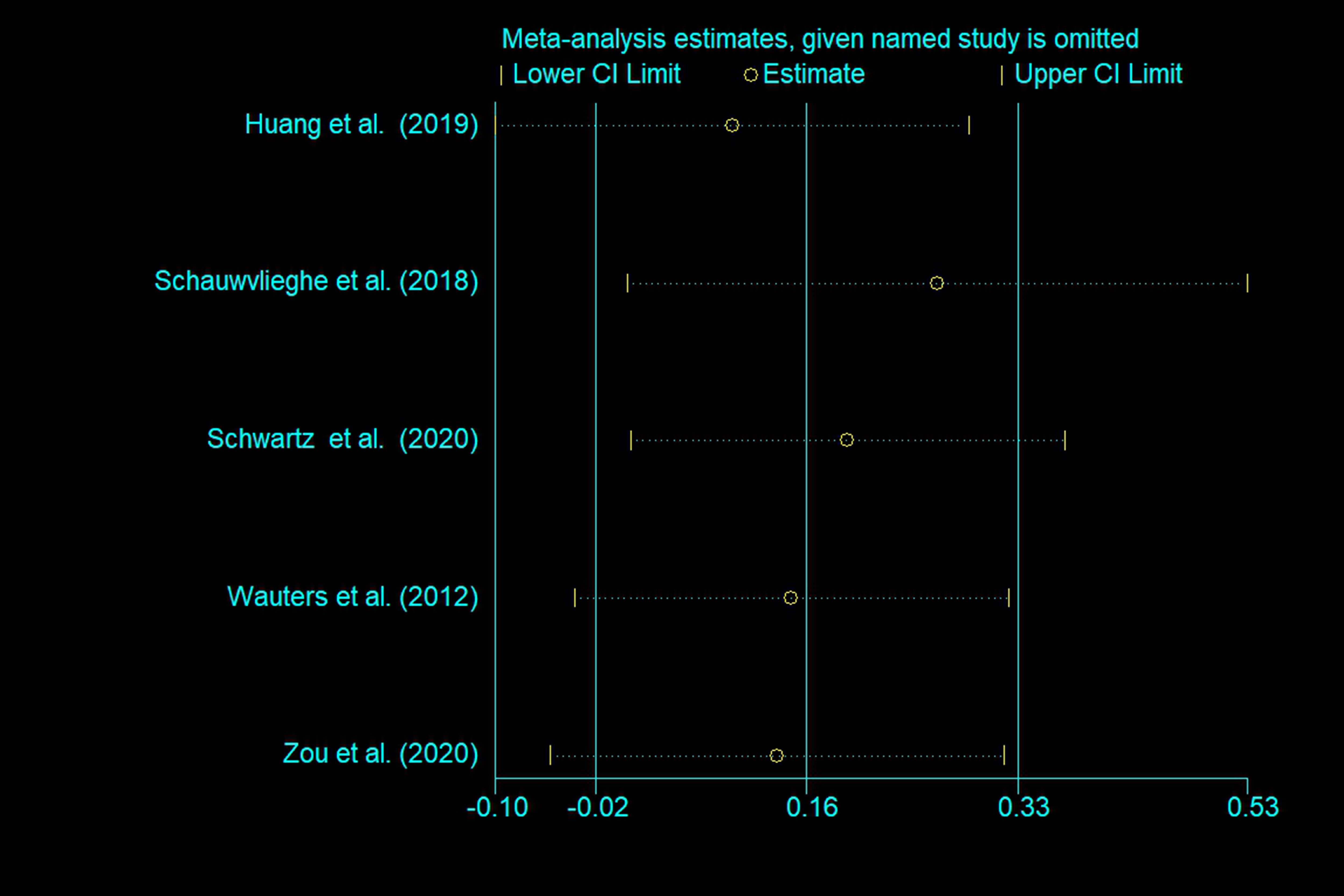

Supplement: Supplementary file 11 — Suppoporting information. [file IID3-11-e760-s003.jpg]

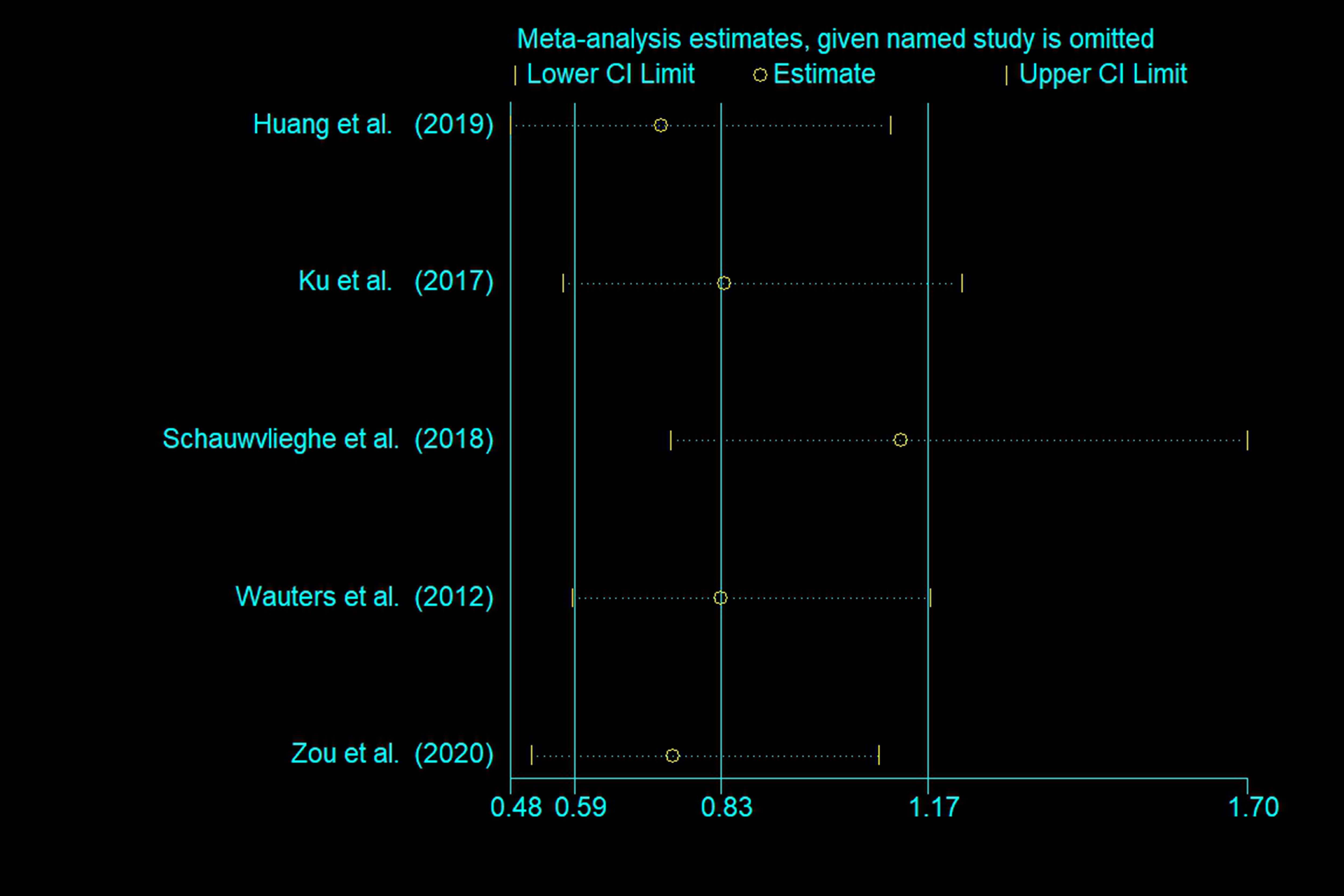

Supplement: Supplementary file 12 — Suppoporting information. [file IID3-11-e760-s001.jpg]

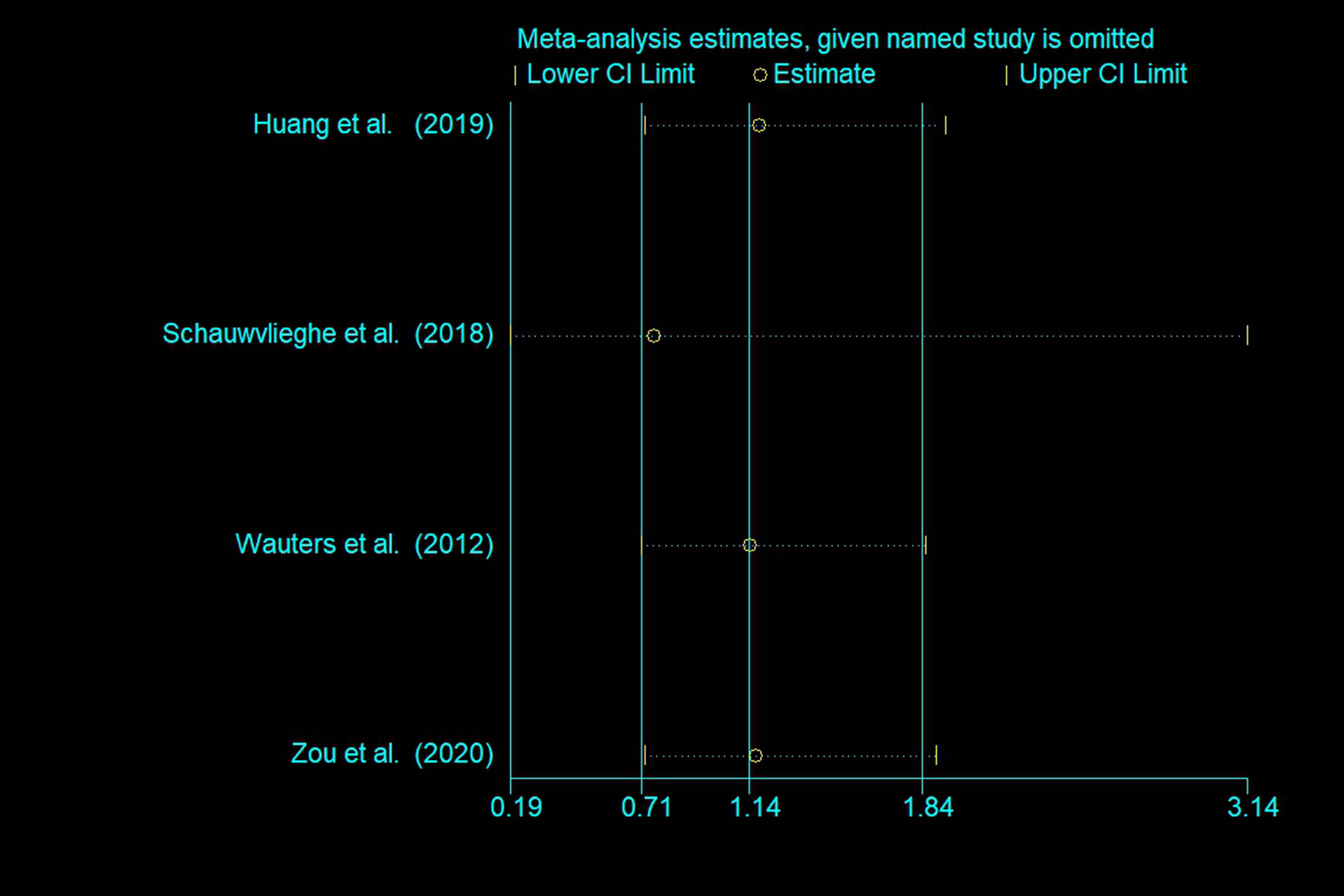

Supplement: Supplementary file 13 — Suppoporting information. [file IID3-11-e760-s004.jpg]

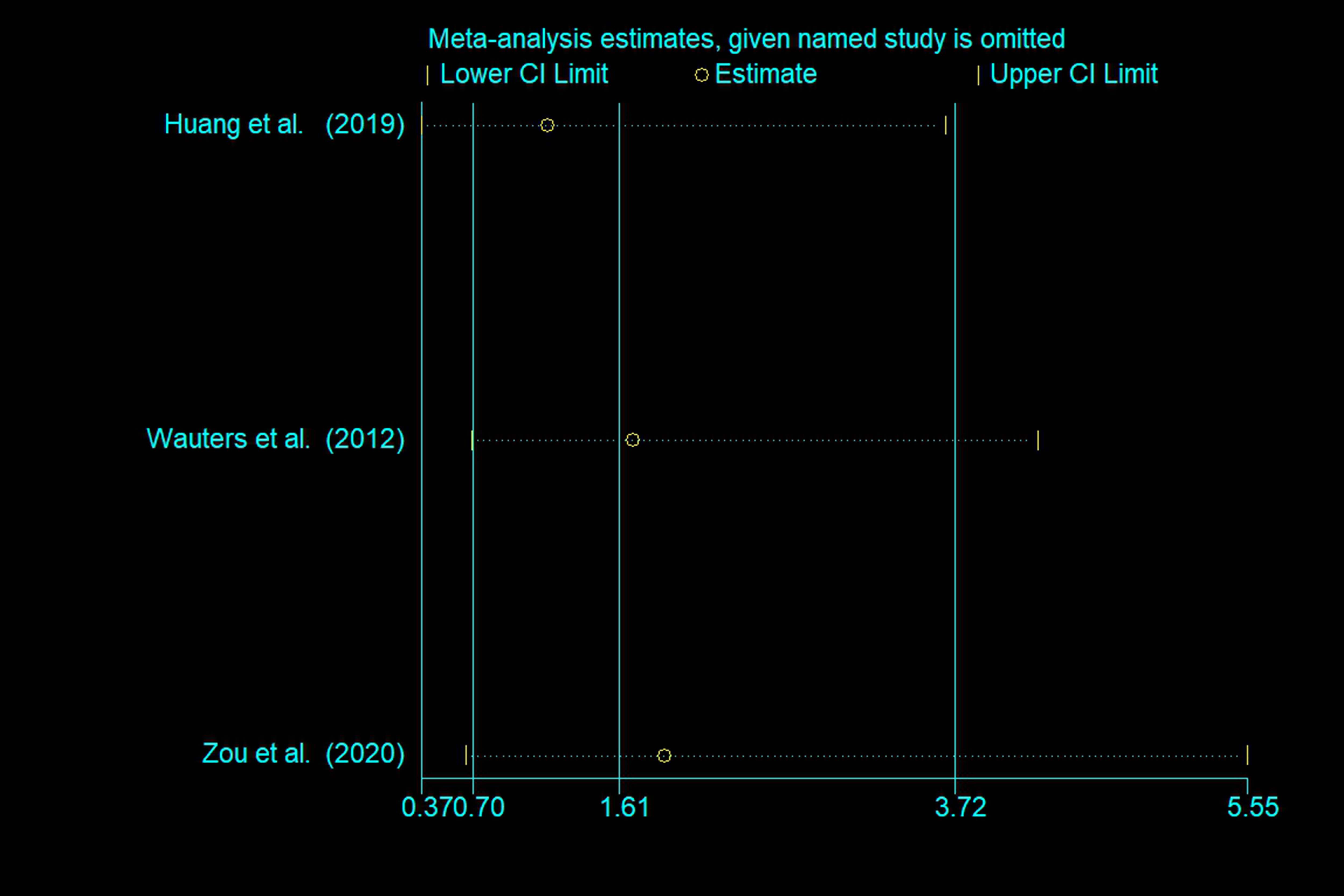

Supplement: Supplementary file 14 — Suppoporting information. [file IID3-11-e760-s006.jpg]
